# Potential causal link between dietary intake and epilepsy: a bidirectional and multivariable Mendelian randomization study

**DOI:** 10.3389/fnut.2024.1451743

**Published:** 2024-08-30

**Authors:** Shenglong Lai, Yazhou Xing, Haiyang Li, Du Wu, Lin Wang, Qinghua Liang

**Affiliations:** Department of Neurosurgery, Henan Provincial People's Hospital, People's Hospital of Zhengzhou University, People's Hospital of Henan University, Zhengzhou, Henan, China

**Keywords:** epilepsy, dietary intake, Mendelian randomization, non-oily fish intake, genome-wide association study

## Abstract

**Background:**

Epilepsy is a common neurological disease, and dietary intake has been suggested as a potential modifiable risk factor. However, the causality of associations between dietary intake and epilepsy remains uncertain. This study aimed to investigate the potential causal relationships between various dietary intakes and epilepsy using Mendelian randomization (MR) analysis.

**Methods:**

A two-sample MR approach was employed, utilizing genetic variants associated with dietary factors as instrumental variables (IVs). Genome-Wide Association Study (GWAS) summary data on dietary intakes were obtained from the UK Biobank, while data on epilepsy were sourced from the European Bioinformatics Institute. The number of genetic variants used as IVs varied for each dietary factor. Inverse-variance weighted (IVW), weighted median, MR-Egger, and Bayesian weighted MR (BWMR) methods were used to assess causality. Multivariable MR (MVMR) was performed, adjusting for potential confounders. Sensitivity analyses were conducted to evaluate the robustness of the findings.

**Results:**

The study identified a significant inverse association between non-oily fish intake and epilepsy risk (OR = 0.281, 95% CI: 0.099–0.801, *p* = 0.018) using the IVW method. This finding was corroborated by the BWMR analysis (OR = 0.277, 95% CI: 0.094–0.814, *p* = 0.020). The MVMR analysis further confirmed the protective effect of non-oily fish intake on epilepsy risk after adjusting for potential confounders. In the reverse MR analysis, epilepsy was associated with reduced water intake (OR = 0.989, 95% CI: 0.980–0.997, *p* = 0.011).

**Conclusion:**

The present MR study provides evidence of a causal, protective relationship between non-oily fish intake and reduced epilepsy risk. Additionally, the findings suggest that epilepsy may influence water intake patterns. These results contribute to our understanding of the role of dietary factors in epilepsy and may inform dietary recommendations for the management and prevention of this condition.

## 1 Introduction

Epilepsy is a chronic neurological disease characterized by recurrent, unprovoked seizures. According to the International League Against Epilepsy (ILAE), epilepsy encompasses a wide spectrum of seizure types and syndromes, each with distinct etiologies, prognoses, and treatment responses (1–3). Globally, epilepsy impacts around 70 million individuals, during a 12-month follow-up period, those experiencing persistent seizures face a risk of 20 to 40% for physical injuries, including fractures, burns, and concussions (4). The Global Burden of Disease Study highlights that the economic impacts of epilepsy-related morbidity and mortality are significant (5, 6). The causes of epilepsy in children and adolescents can vary widely and include genetic factors, structural abnormalities, metabolic disorders, and unknown etiologies (7). Antiseizure medications (ASMs) are classified based on their mechanism of action and efficacy in treating different types of seizures (8). However, they do not address the underlying predisposition to seizures. Currently, available ASMs are effective in approximately two-thirds of epilepsy patients (9). Neurosurgical resection is an effective strategy for achieving seizure control in selected individuals with drug-resistant focal epilepsy (10, 11). Non-pharmacological treatments, including palliative surgery, neuromodulation techniques, and dietary interventions, offer therapeutic options for patients with drug-resistant epilepsy who are not candidates for resective brain surgery (2). Although multiple theories have been proposed to elucidate the mechanisms behind epilepsy, the precise causes and risk factors for the condition remain unknown (12). Thus, the quest for and identification of modifiable risk factors to preclude the onset of epilepsy holds paramount significance.

Numerous factors are regarded as potential triggers for seizures (2). At present irregularities in the dietary habits of the people with epilepsy have been identified (13–17). Dietary habits are a primary cause of variations in the gut microbiome (18). Growing evidence has underscored the crucial role of the microbiota-gut-brain (MGB) axis in epilepsy, suggesting that non-pharmacological interventions targeting the gut microbiota could serve as potential preventive and therapeutic approaches (19). Specifically, restoring intestinal eubiosis may help reduce the onset of seizures by modulating mechanisms related to epilepsy. In a clinical trial involving 45 patients with drug-resistant epilepsy, a probiotic mixture intake reduced seizure frequency by 50% or more in 28.9% of participants (20). Furthermore, the ketogenic diet (KD), characterized by a very low carbohydrate content and a high-fat content, has also attracted attention for its potential to influence the composition of the gut microbiota, thereby impacting the treatment of epilepsy (21). Previous observational studies have reported associations between various dietary intakes and epilepsy (13). However, the causality of these associations remains uncertain. Therefore, the present study was designed to investigate the potential causal relationships between dietary intake and epilepsy.

Randomized Controlled Trials (RCTs) are considered the gold standard for testing causal relationships between exposures and outcomes (22), however, they require significant human and material resources. Currently, Mendelian Randomization (MR) is an epidemiological method similar to RCTs and is widely used to determine the causal relationships between risk factors and disease outcomes (23). MR uses genetic instrumental variables (IVs) to evaluate associations between exposures and outcomes, minimizing the impact of confounding factors and potentially strengthening the robustness of the established causal relationships. In this study, we performed a two-sample MR analysis to explore the causal relationship between various dietary intakes and epilepsy. To provide a balanced discussion, we acknowledge the limitations of Mendelian Randomization (MR). MR relies on several key assumptions: the genetic instruments must be robustly associated with the exposure, must not be associated with any confounders, and must influence the outcome only through the exposure. Potential limitations include the need for robust validation of genetic instruments to ensure they meet these assumptions. Moreover, MR studies must ensure that the instruments are not pleiotropic, which means the genetic variants should not affect the outcome through pathways other than the exposure of interest. These limitations underline the importance of careful instrument selection and validation in MR studies to ensure reliable causal inferences.

## 2 Materials and methods

### 2.1 Study design

**Figure 1:**
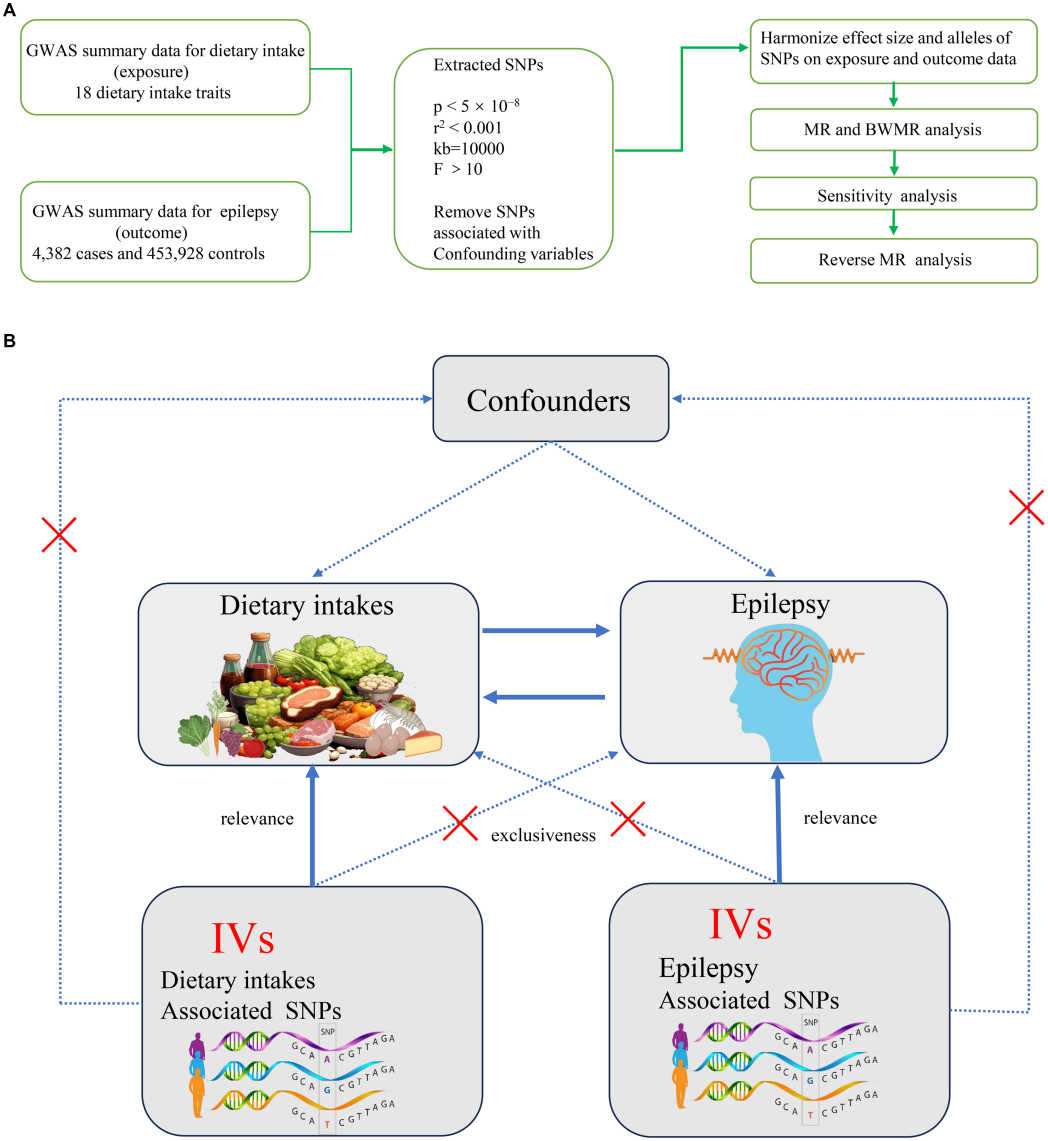
**(A)** Overall flow chart of this study. **(B)** Assumptions and study design of the MR study of the associations between 18 dietary intakes and epilepsy. GWAS, Genome-Wide Association Study; MR, Mendelian randomization; BWMR, Bayesian weighted mendelian randomization; SNPs, single-nucleotide polymorphisms.

As detailed in the Data Source section, our study included a total of 4,382 epilepsy cases and 453,928 controls sourced from the European Bioinformatics Institute (EBI) database. The current study is the first to utilize MR design to explore the causal relationship between various dietary intakes and epilepsy. Figure 1A depicts the overall design of present MR study. Initially, we employed a two-sample MR approach to investigate the causal relationships between various dietary factors and epilepsy. To ensure the reliability of MR findings, our study aimed to meet the following three assumptions: First, genetic IVs must be strongly associated with dietary intake (Assumption 1). Second, the selected genetic IVs should not be correlated with potential confounding factors (Assumption 2). Third, the chosen IVs should not independently influence the occurrence of epilepsy (Assumption 3) (Figure 1B). We employed several MR methods to assess the causal relationships between dietary intakes and epilepsy. The Inverse Variance Weighted (IVW) method is the primary approach, which estimates causal effects by averaging the effect estimates from each genetic variant, weighted by their precision. This method assumes that all genetic variants are valid instruments and that they are independent of each other. In contrast, the Bayesian Weighted Mendelian Randomization (BWMR) method incorporates a prior distribution to account for potential pleiotropy, which is the phenomenon where genetic variants affect the outcome through multiple pathways. BWMR provides a more nuanced understanding by adjusting the weights of the genetic instruments based on their pleiotropic effects. The Multivariable Mendelian Randomization (MVMR) method extends the MR analysis by adjusting for potential confounders. It allows for the estimation of the direct effects of dietary intake on epilepsy while controlling for other variables that may influence the relationship. MVMR is particularly useful when dealing with multiple exposures and potential confounders.

### 2.2 Data source

The dietary factors included in the study encompassed various food and beverage categories, such as meat intake (processed meat, beef, pork, lamb/mutton, oily fish, non-oily fish, and poultry), drink intake (water, tea, coffee, and alcoholic drinks frequency), staple diet intake (bread and cereal), dairy product intake (cheese), and fruit and vegetable intake (dried fruit, fresh fruit, cooked vegetables and salad/raw vegetables). The present study aimed to analyze the potential impacts of these dietary factors on the epilepsy.

The Genome-Wide Association Study (GWAS) data for various dietary intake factors were extracted from the UK Biobank. Additionally, we sourced GWAS summary data on epilepsy, which included genotype information for 4,382 epilepsy patients and 453,928 controls, from the EBI database. The specific information on the data can be found in Table 1. The summary data of both GWAS analyses were derived from IEU Open GWAS Project.^1^ All GWAS datasets used in present MR study are based on publicly available summary data, hence moral approval and participant consent are not required.

**Table 1:** The Genome-Wide Association Study (GWAS) data for dietary intakes and epilepsy.

Exposure or outcome	GWAS ID	Sample size	Identified SNPs
Processed meat intake	ukb-b-6324	461,981	23
Beef intake	ukb-b-2862	461,053	17
Pork intake	ukb-b-5640	460,162	14
Lamb/mutton intake	ukb-b-14179	460,006	32
Non-oily fish intake	ukb-b-17627	460,880	11
oily fish intake	ukb-b-2209	460,443	64
Poultry intake	ukb-b-8006	461,900	8
Water intake	ukb-b-14898	427,588	42
Tea intake	ukb-b-6066	447,485	41
Coffee intake	ukb-b-5237	428,860	43
Alcohol intake frequency	ukb-b-5779	462,346	101
Bread intake	ukb-b-11348	452,236	32
Cereal intake	ukb-b-15926	441,640	43
Cheese intake	ukb-b-1489	451,486	65
Dried fruit intake	ukb-b-16576	421,764	43
Fresh fruit intake	ukb-b-3881	446,462	55
Cooked vegetable intake	ukb-b-8089	448,651	17
Salad/raw vegetable intake	ukb-b-1996	435,435	24
Epilepsy	ebi-a-GCST90018840	458,310	NA

### 2.3 Selection of IVs

To ensure both robustness and an adequate number of IVs, we carefully selected scientifically validated single nucleotide polymorphisms (SNPs) as IVs to assess the causal relationship between dietary intake and epilepsy. We applied a strict threshold of *p* < 5 × 10^−8^ to ensure the inclusion of a sufficient number of SNPs while maintaining the statistical integrity of the results. Applying *r*^2^ < 0.001 and choosing a broader region size (10,000 kb) as the IVs selection criteria achieves a dual purpose, it minimizes the correlation between IVs and the target locus while mitigating potential confounding due to linkage disequilibrium (LD). To mitigate bias caused by weak IVs, a critical measure is to ensure that the F-statistic exceeds 10, thereby confirming the validity of the IV and ensuring robust causal inference. Furthermore, palindromic SNPs were excluded from the selection of IVs. Additionally, the removal of confounders from IVs, essential for MR analysis, was facilitated using the LDtrait Tool.^2^ LDtrait Tool is a web-based application that assesses linkage disequilibrium (LD) and trait associations of SNPs to identify and exclude potential confounders. Finally, to address potential horizontal pleiotropy, MR pleiotropy residual sum and outlier (MR-PRESSO) tests were conducted. MR-PRESSO is a method that detects and corrects for horizontal pleiotropy by identifying outliers and providing adjusted causal estimates. Any outliers identified during these tests were excluded to minimize the influence of pleiotropy. Subsequently, in conducting reverse MR analysis with epilepsy as the exposure and various dietary intakes as outcomes, the selection of IVs was required to meet the previously outlined four criteria. Due to the substantial number of IVs obtained when using epilepsy as the exposure, we implemented stricter screening criteria for the correlation hypothesis, setting a threshold of *p* < 5 × 10^−6^.

### 2.4 Methodology of MR

Various MR methods were employed to deduce causal relationships between different dietary intakes and epilepsy. The Inverse Variance Weighted (IVW) method, which comprehensively accounts for the effects of each SNP by weighted averaging of effect estimates from numerous genetic variants, served as the primary approach for estimating causal effects in this study. In contrast, the Wald Ratio method was used as a complementary tool to evaluate the effect of individual SNP. The Weighted Median and MR-Egger methods work synergistically to enhance analyses, each excelling under different conditions. The weighted median approach reduces the influence of extreme effects amidst heterogeneity. MR-Egger, which does not require uniform directions of action for all IVs, suits skewed situations, thereby limiting the added impact of genetic variation on exposure-outcome relationships.

### 2.5 BWMR

BWMR was applied to account for pleiotropy and enhance the robustness of causal estimates. This method incorporates prior knowledge and updates the weights of genetic instruments, providing more accurate and reliable results in assessing the effect of dietary intake on epilepsy (24).

### 2.6 MVMR

In this study, we used MVMR to evaluate the causal relationship between dietary intake and epilepsy. We employed genetic variants associated with dietary factors as IVs while controlling for potential confounders, such as brain tumor (GWAS ID: ebi-a-GCST90018800), intracerebral hemorrhage (GWAS ID: ebi-a-GCST90018870), and diffuse brain injury (GWAS ID: finn-b-ST19_DIFFU_BRAIN_INJURY). This method allows for the estimation of the direct effects of dietary intake on epilepsy, taking into account these confounding factors.

### 2.7 Sensitivity analysis

In two samples MR analysis, assessments of heterogeneity and pleiotropy were conducted. The Cochran’s IVW Q statistics were utilized to evaluate the variability among the IVs. In cases where variation in the estimated effects was observed, we transitioned from the standard fixed effects IVW method to the random effects IVW technique to accommodate this diversity.

The MR-Egger method was used with caution to detect the presence of horizontal pleiotropy. When the MR-Egger intercept was found to be statistically significant, it indicated that the association findings might be confounded by horizontal pleiotropic effects of other traits. To exclude possible horizontal pleiotropy, the global test MR-PRESSO (MR pleiotropy residual sum and outlier) was also performed to determine if there were any outliers. In addition, each instrumental SNP was omitted in turn as part of a leave-one-out analysis to identify potentially heterogeneous SNPs. The scatter plots showed that the results were not affected by anomalies, while the leave-one-out plots showed the robustness of the association.

### 2.8 Statistical analysis

The presentation of odds ratios (OR) and 95% confidence intervals (CI) in the statistical results indicates significance when *p* < 0.05. Analyses were performed using R 4.4.0 software. The packages “TwoSampleMR” and “MendelianRandomization” in R were utilized for MR analyses and clumping. Finally, MR-PRESSO was performed by the MR-PRESSO package.

## 3 Results

### 3.1 Selection of instrumental variables

We analyzed the causal associations of dietary intake factors with epilepsy across 18 different exposures. The number of SNPs used in our study ranged from 8 to 101. The F-statistics for all IVs exceeded 10, indicating strong associations with the exposures. Compared to the exposure datasets, there was minimal potential for population stratification bias. Further details are provided in Supplementary Table S1. Additionally, due to non-significant conclusions from the MR-PRESSO global test (*p* > 0.05), no outliers were removed through MR-PRESSO analysis.

### 3.2 MR and BWMR analysis of dietary intake factors on epilepsy

**Figure 2:**
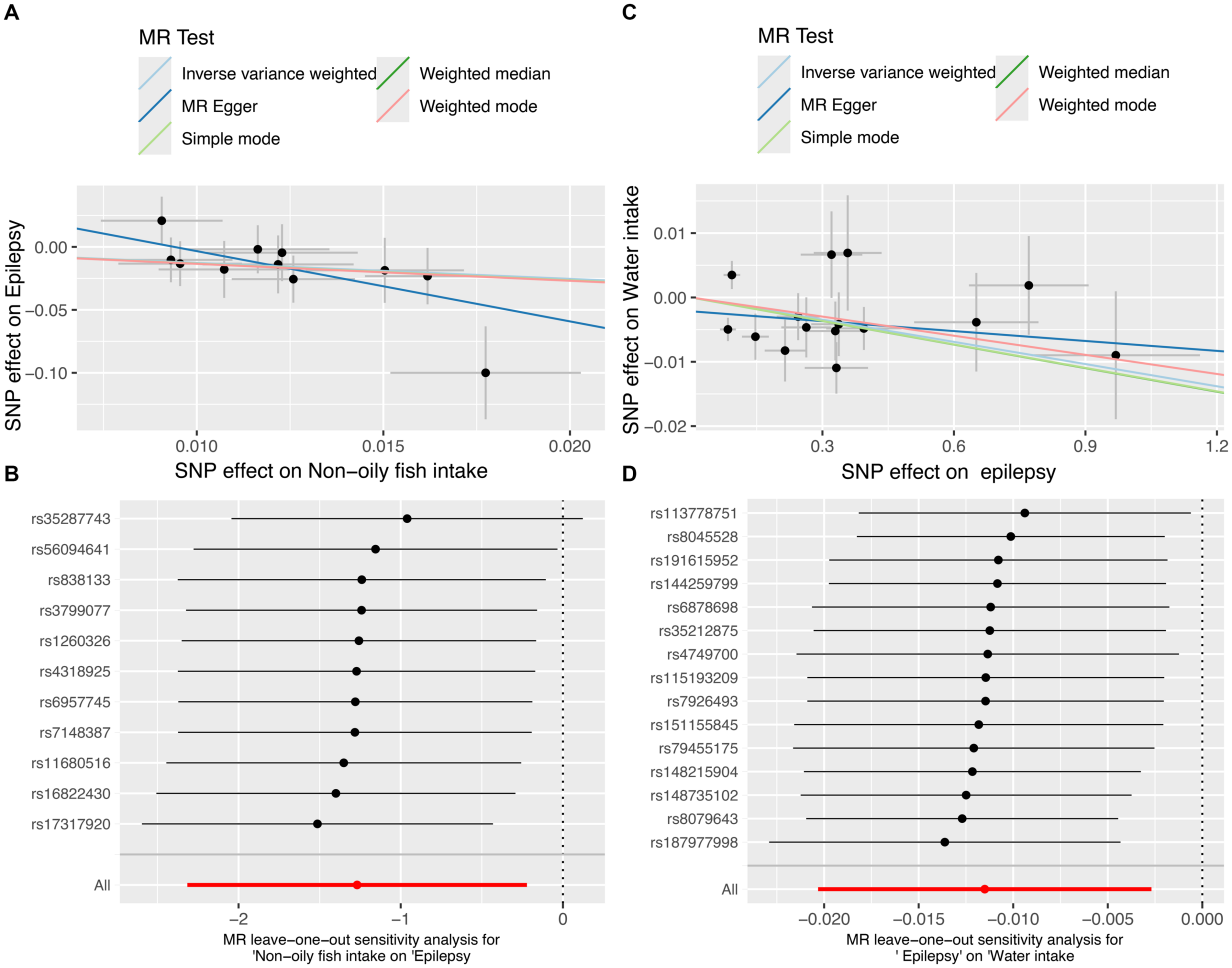
The MR test for non-oily fish intake on epilepsy and the reverse MR test for epilepsy on water intake. **(A)** Scatter plot of MR analysis for non-oily fish intake on epilepsy. **(B)** MR leave-one out sensitivity analysis for non-oily fish intake on epilepsy. **(C)** Scatter plot of MR analysis for epilepsy on water intake. **(D)** MR leave-one out sensitivity analysis for epilepsy on water intake. MR, mendelian randomization; SNPs, single-nucleotide polymorphisms.

**Figure 3:**
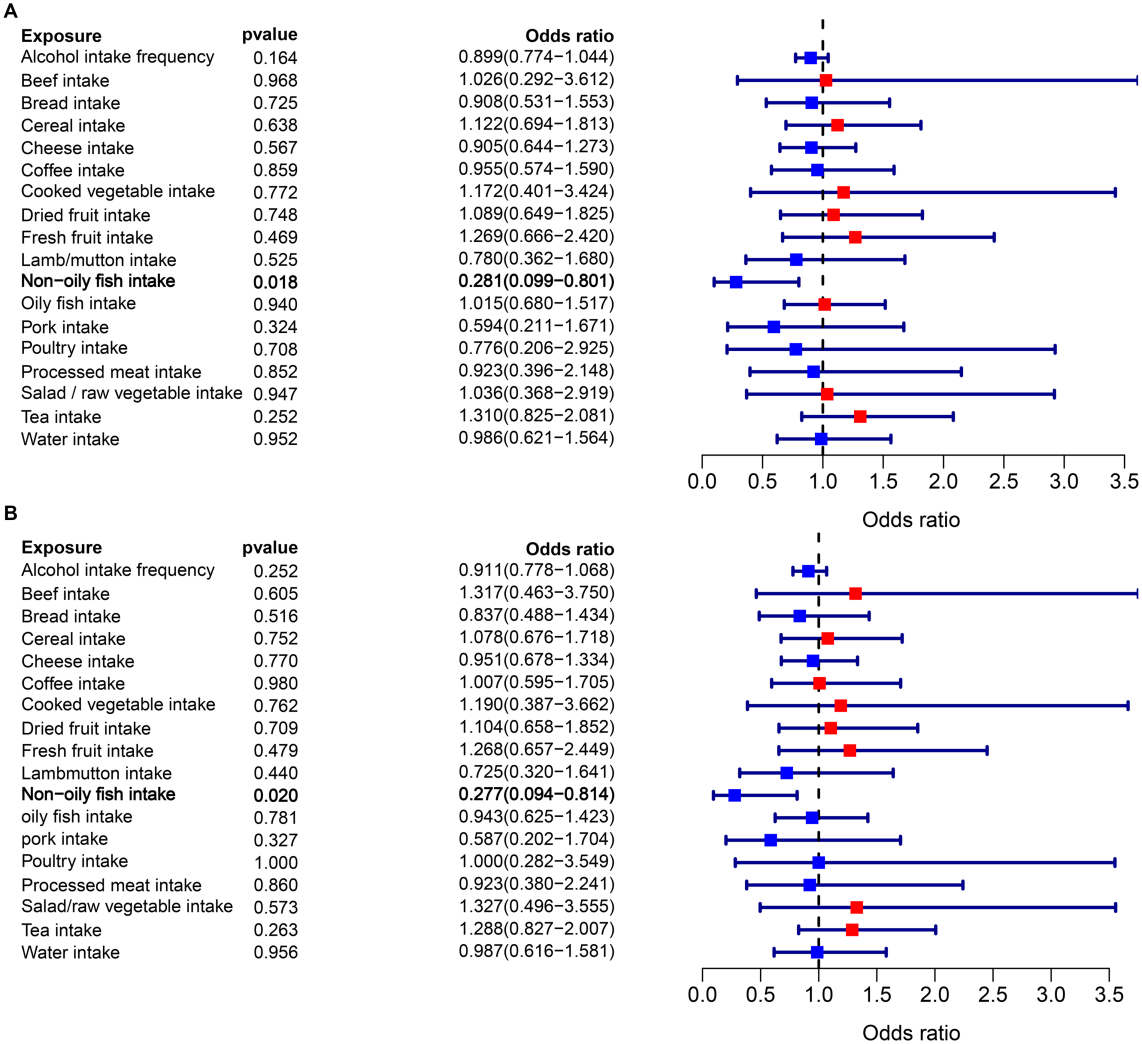
**(A)** Forest plots of the MR results (IVW method) to present the causal associations of 18 dietary intake factors on epilepsy risk. **(B)** Forest plots of the MR results (BW method) to present the causal associations of 18 dietary intake factors on epilepsy risk.

In the MR analysis, one dietary intake was found to be causally associated with the risk of epilepsy. Notably, using the IVW method as the primary analytical approach, the intake of non-oily fish (OR = 0.281, 95% CI = 0.099–0.801, *p* = 0.018) exhibited a significant association with lower odds of epilepsy (Figure 2A). While factors such as alcohol intake (OR = 0.899, 95% CI = 0.774–1.044, *p* = 0.164), coffee intake (OR = 1.122, 95% CI = 0.694–1.813, *p* = 0.638), which were previously thought to play a negative role in epilepsy, do not have a significant causal relationship with epilepsy. Detailed analysis results can be found in Figure 3A, Supplementary Figure S2, and Supplementary Table S2.

Moreover, the findings from the BWMR analysis supported the previous MR results, indicating a significant inverse association between non-oily fish intake and the likelihood of epilepsy (OR = 0.277, 95% CI = 0.094–0.814, *p* = 0.020). This association is illustrated in Figure 3B and Supplementary Table S3.

### 3.3 Reverse MR and BWMR analysis of epilepsy on dietary intake factors

**Figure 4:**
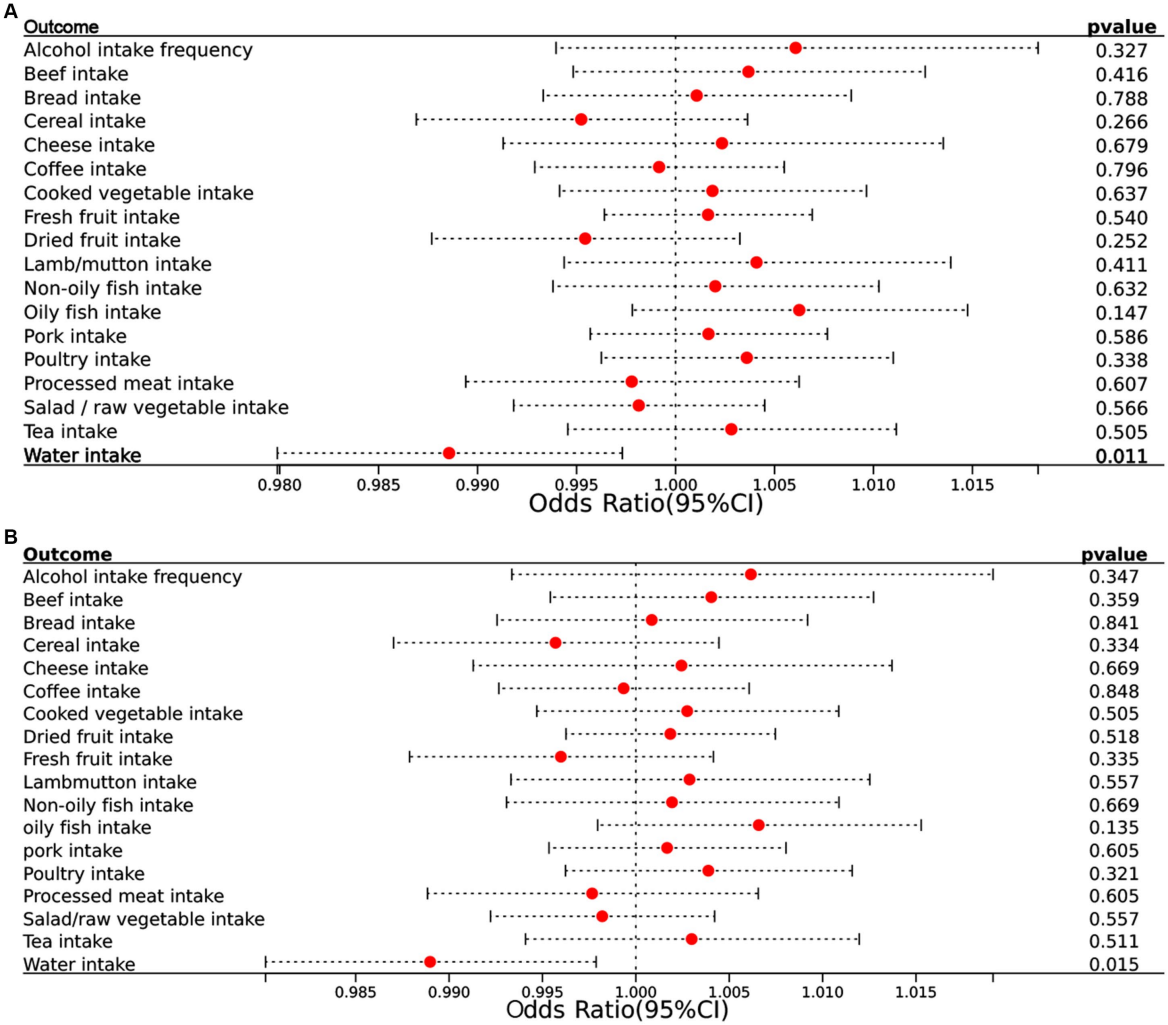
**(A)** Forest plots of the reverse MR results (IVW method) to present the causal associations of epilepsy on 18 dietary intake factors. **(B)** Forest plots of the reverse MR results (BW method) to present the causal associations of epilepsy on 18 dietary intake factors. CI, confidence interval.

In the reverse MR analysis, where epilepsy was treated as the exposure and dietary intake factors as the outcomes, we observed an inverse association between epilepsy and water intake (OR = 0.989, 95% CI = 0.980–0.997, *p* = 0.011) (Figure 2C). This suggests that individuals with epilepsy may have a slightly reduce of water intake. MR analysis showed negative results for epilepsy on the remaining dietary intake factors. The results show in Figure 4A.

BWMR confirmed these findings with consistent results (OR = 0.989, 95% CI = 0.980–0.998, *p* = 0.015), further validating the accuracy of our results. The accompanying Figure 4B illustrates these associations clearly.

### 3.4 MVMR analysis of dietary intake factors on epilepsy

**Figure 5:**
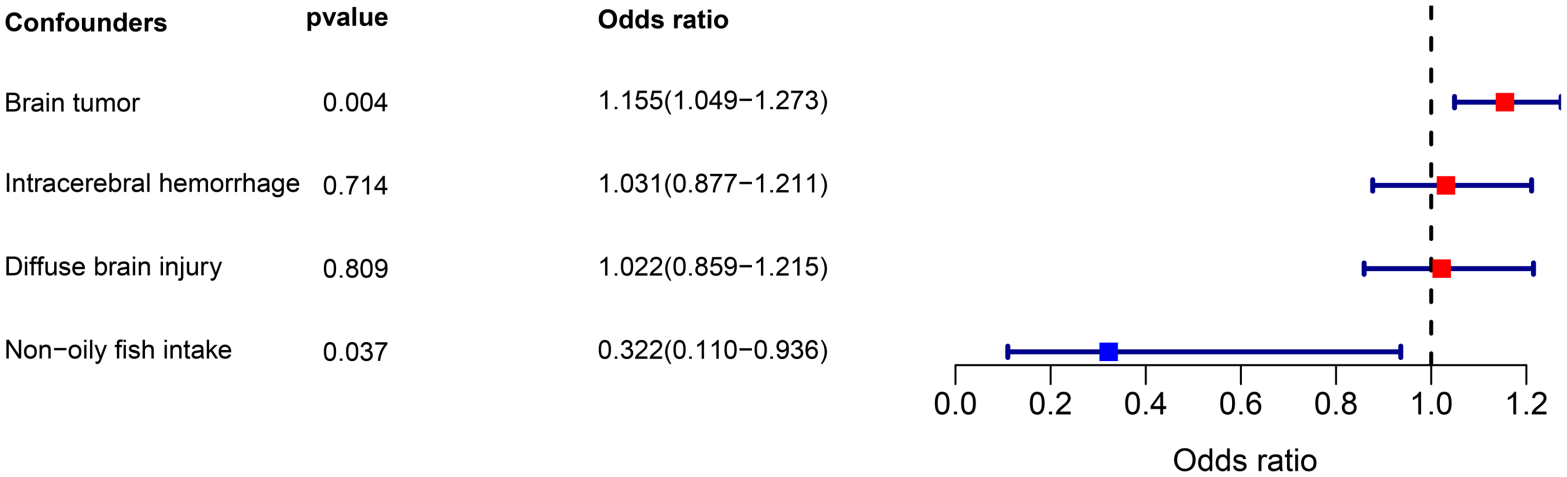
Results of multivariable MR analysis for dietary intake factors on epilepsy.

The MVMR analysis included the positive factor non-oily fish intake and three potential confounders: brain tumor, intracerebral hemorrhage, and diffuse brain injury. The results of the MVMR analysis showed that non-oily fish intake was significantly associated with a lower risk of epilepsy (OR = 0.322, 95% CI = 0.110–0.936, *p* = 0.037). Additionally, we observed that brain tumor was positively associated with epilepsy risk (OR = 1.155, 95% CI = 1.049–1.273, *p* = 0.004), while there was no significant association found for intracerebral hemorrhage (OR = 1.031, 95% CI = 0.877–1.211, *p* = 0.714) and diffuse brain injury (OR = 1.022, 95% CI = 0.859–1.215, *p* = 0.809). The results of the forest plot are depicted in Figure 5.

### 3.5 Sensitivity analysis

Additionally, Cochran’s Q tests revealed no heterogeneity (*p* > 0.05 for all outcomes), except for the causal association between tea intake and epilepsy. The MR-Egger intercept test showed no statistically significant evidence of horizontal pleiotropy in the study. Leave-one-out analyses indicated that no single SNP significantly influenced the MR findings. Collectively, the sensitivity analyses bolstered the reliability of our results. Detailed information can be found in Supplementary Tables S4–S6 and Supplementary Figures S1–S4.

## 4 Discussion

The present study describes the causal relationship between genetically predicted dietary intake and epilepsy. We found that increased intake of non-oily fish decreased the odds of developing epilepsy. Therefore, we are contemplating the examination of whether supplementing scientific dietary intake choices, in addition to the existing management for epilepsy patients, could potentially reduce the odds of epilepsy progression. Understanding these relationships is crucial for developing dietary recommendations for the management and prevention of epilepsy.

While there is extensive literature on the health benefits of oily fish intake (25–27), research on the relationship between non-oily fish and epilepsy is limited. Non-oily fish intake has garnered attention in the study of the relationship between dietary intake and epilepsy due to its potential protective effects. Non-oily fish, which includes varieties such as cod, haddock, and tilapia, is known for its low fat content and high nutritional value, particularly in omega-3 fatty acids. Our Mendelian randomization study further substantiates the potential protective role of non-oily fish intake against epilepsy. By utilizing genetic variants associated with non-oily fish intake as instrumental variables, we found a significant and causal association between higher non-oily fish intake and a lower risk of epilepsy. This provides robust evidence supporting the notion that including non-oily fish in one’s diet may serve as a protective factor against epilepsy. The underlying reasons for the observed protective effect of non-oily fish intake on epilepsy warrant further investigation. Compared to oily fish, non-oily fish is lower in fat, higher in protein, easier to digest, and a good source of ω-3 polyunsaturated fatty acids, vitamins B12, B6. Previously published data suggests that Docosahexaenoic Acid (DHA) supplementation as an add-on treatment may help reduce seizure frequency in canine idiopathic epilepsy (28). Additionally, omega-3 supplementation has shown beneficial effects on seizure frequency in both adults and children with epilepsy (29). However, long-term treatment with certain older ASMs may lead to hyperhomocysteinemia (30). B-vitamins have been shown to reduce total homocysteine concentrations in patients undergoing chronic treatment with ASMs (31), though the most appropriate supplementation regimen for B-vitamins in epilepsy patients remains debated (32). This is partly because the blood–brain barrier limits the passage of vitamins into the central nervous system, making excessive supplementation potentially harmful. Vitamins possess antioxidant, anti-inflammatory, and immunomodulatory properties (33). Researches have reported the efficacy of Vitamin C and E in reducing seizure frequency among drug-resistant epileptic patients (34, 35). These findings, in conjunction with the established antioxidant properties of vitamins, support the potential therapeutic role of certain micronutrients in epilepsy management. Supplementing with vitamins can be beneficial for treating epilepsy, and non-oily fish may be a good dietary choice to provide these nutrients. Furthermore, oily fish can accumulate higher levels of toxins such as mercury and polychlorinated biphenyls, which can negatively impact brain development and function (36).

The relationship between dietary intake and epilepsy has been the focus of many previous studies, with various factors proposed as potential risk factors for epilepsy (37). We acknowledge that our findings regarding certain dietary factors, specifically coffee and alcohol intake (38–41), do not indicate a clear causal relationship with epilepsy risk. This observation may appear to contradict some previous studies that have suggested potential associations between these dietary habits and epilepsy. However, it is essential to consider the methodological differences between these studies and ours. Unlike traditional observational studies that are susceptible to confounding factors and reverse causality, our Mendelian Randomization (MR) approach utilizes genetic variants as instrumental variables to provide less biased estimates of causality. The discrepancy between our findings and some previous literature may also be attributed to the specific populations studied, the accuracy of dietary intake measurements, and the potential for residual confounding factors that are not accounted for in non-MR studies. It is also plausible that the relationship between these dietary factors and epilepsy is more complex than initially thought and may vary across different populations and settings. Our findings contribute to the growing body of evidence that challenges the previously established beliefs about the role of coffee and alcohol in epilepsy risk. While our study does not support a causal effect of these factors, it is crucial to continue investigating their potential indirect effects or interactions with other risk factors in epilepsy development.

In light of these considerations, our results should be interpreted with caution, and further research is warranted to explore the complex interplay between diet, lifestyle, and epilepsy risk. Future studies may benefit from employing MR designs to strengthen causal inferences and from incorporating diverse populations to enhance the generalizability of the findings. Moreover, the observed heterogeneity in the association between tea intake and epilepsy could be attributed to variations in tea consumption patterns, tea types, or other lifestyle factors that were not adequately captured in our research. This heterogeneity highlights the complexity of dietary influences on the risk of epilepsy and suggests that a more nuanced approach is necessary to fully comprehend these relationships.

Interestingly, in our reverse MR analysis, where epilepsy was treated as the exposure and dietary intake factors as the outcomes, we identified a significant association indicating that epilepsy influences water intake. This finding was corroborated by BWMR analysis, which produced consistent results, further supporting the robustness of this association. Previous research on the relationship between epilepsy and water intake is limited, but some studies suggest that patients with epilepsy may experience alterations in their thirst perception and fluid regulation. This could be due to the effects of ASMs (42, 43) or the impact of seizures on the hypothalamus (44), which plays a critical role in maintaining fluid balance. The precise mechanisms underlying the influence of epilepsy on water intake remain unclear and warrant further investigation. It is possible that the neurological changes associated with epilepsy affect the regulatory pathways controlling thirst and fluid balance (45, 46). Additionally, lifestyle factors and comorbid conditions prevalent in individuals with epilepsy might also contribute to changes in water intake patterns (47). Understanding the influence of epilepsy on water intake is crucial, as adequate hydration is essential for overall health and can impact seizure control and quality of life in epilepsy patients. Further studies are needed to elucidate the underlying mechanisms and to explore potential interventions that could help manage water intake in this population.

In addition to our findings on dietary intake, the MVMR analysis identified a positive correlation between brain tumors and the occurrence of epilepsy. Brain tumors can disrupt normal brain function and contribute to epileptogenesis through mechanisms such as increased intracranial pressure, localized tissue damage, and alterations in neuronal activity (48, 49). However, no significant associations were found for intracerebral hemorrhage and diffuse brain injury. The lack of significant association for these conditions might be due to the variability in the extent and location of brain injuries, which influences their potential to cause epilepsy. These findings underscore the complex interplay between neurological conditions and epilepsy, highlighting the need for further research to develop targeted interventions. Addressing underlying neurological disorders is crucial in the comprehensive management of epilepsy.

While our research highlights the protective effects of non-oily fish intake against epilepsy, there are several limitations to consider. Our analysis was confined to 18 dietary factors, potentially overlooking other components that could influence epilepsy. Additionally, the study was conducted on a specific demographic, and its findings may not extend to other groups. Given the diverse causes and types of epilepsy—ranging from genetic factors to brain injuries, and many with unknown origins—our study’s scope might not encompass all variants of the disease. This diversity in etiology limits the applicability of our results across all epilepsy subtypes. Future studies should address this variability to offer more personalized insights and treatment strategies. Moreover, there is a potential for horizontal pleiotropy that could influence the relationship between the genetic variants used as IVs and epilepsy outcomes. Although we employed MR-PRESSO tests to explore this issue, the possibility of unmeasured pleiotropy remains. Furthermore, our findings are based on summary data from specific cohorts, which may limit their generalizability to other populations. Therefore, future research should aim to replicate our findings in different populations and explore the mechanisms underlying the observed associations. Cohort studies with detailed dietary assessments and clinical trials evaluating the impact of specific dietary interventions on epilepsy outcomes are warranted. Additionally, exploring the interaction between genetic factors, diet, and epilepsy may provide further insights into personalized dietary recommendations for epilepsy patients.

In conclution, the present MR investigation has identified non-oily fish intake as protective factors against epilepsy in terms of dietary intake. Additionally, the findings suggest that epilepsy may influence water intake patterns. The integration of findings across multiple study types to further minimize study bias is of significant public health implication in establishing robust evidence of causal associations between dietary intake factors and epilepsy, providing informative support for primary prevention policies of epilepsy. Based on our findings, clinicians may consider recommending dietary adjustments, particularly increasing non-oily fish intake, as part of a comprehensive epilepsy management plan. However, it is important to individualize dietary recommendations based on patient-specific factors and preferences.

## Data Availability

The datasets presented in this study can be found in online repositories. The names of the repository/repositories and accession number(s) can be found in the article/Supplementary material.

## Footnotes

^1^ https://gwas.mrcieu.ac.uk

^2^ https://ldlink.nih.gov/?tab=ldtrait

## Supplementary material

Supplementary Figure S1 MR leave-one out sensitivity analysis for dietary intake factors on epilepsy.

Supplementary Figure S2 Scatter plot of MR analysis for dietary intake factors on epilepsy.

Supplementary Figure S3 MR effect size for dietary intake factors on epilepsy.

Supplementary Figure S4 Funnel plot from single SNP analyses for dietary intake factors on epilepsy.

## Glossary

MR: Mendelian randomization
IVs: Instrumental variables
GWAS: Genome-Wide Association Study
IVW: Inverse-variance weighted
BWMR: Bayesian weighted Mendelian randomization
MVMR: Multivariable MR
LD: Linkage disequilibrium
MR-PRESSO: MR-pleiotropy residual sum and outlier
ID: Identification
OR: Odds ratio
CI: Confidence interval
P: p-value
RCTs: Randomized Controlled Trials
MGB: Microbiota-gut-brain
KD: Ketogenic diet
EBI: European Bioinformatics Institute
DHA: Docosahexaenoic Acid
ILAE: International League Against Epilepsy
ASMs: Antiseizure medications
