# Supplementary figures and images for: Potential causal link between dietary intake and epilepsy: a bidirectional and multivariable Mendelian randomization study
Source: Front Nutr. 2024 Aug 30;11:1451743. doi: 10.3389/fnut.2024.1451743 (PMC11392887; doi:10.3389/fnut.2024.1451743)

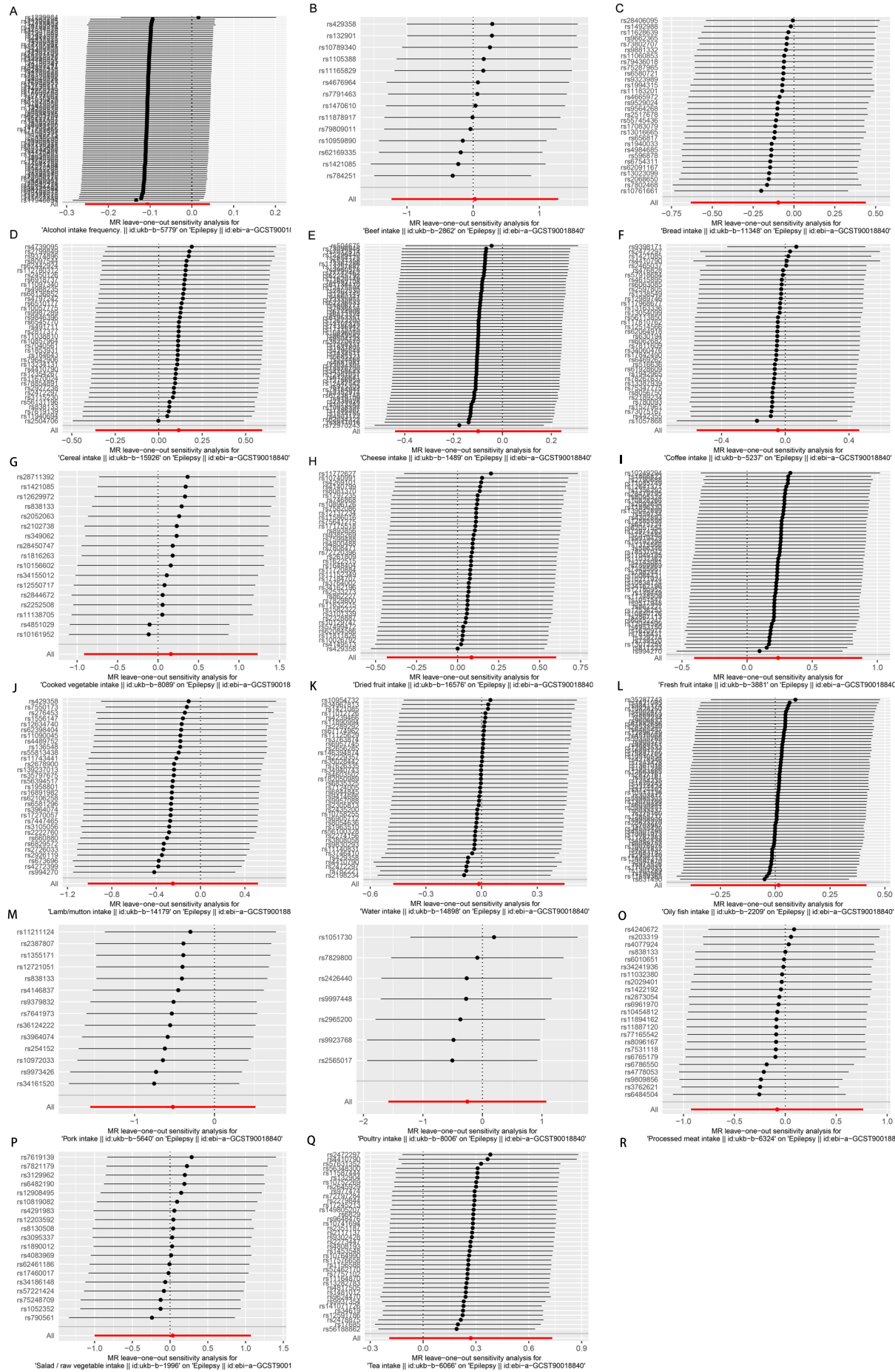

Supplement: Supplementary Figure S1 — MR leave-one out sensitivity analysis for dietary intake factors on epilepsy. [file Data_Sheet_2.zip › Supplementary Figure S1.pdf]

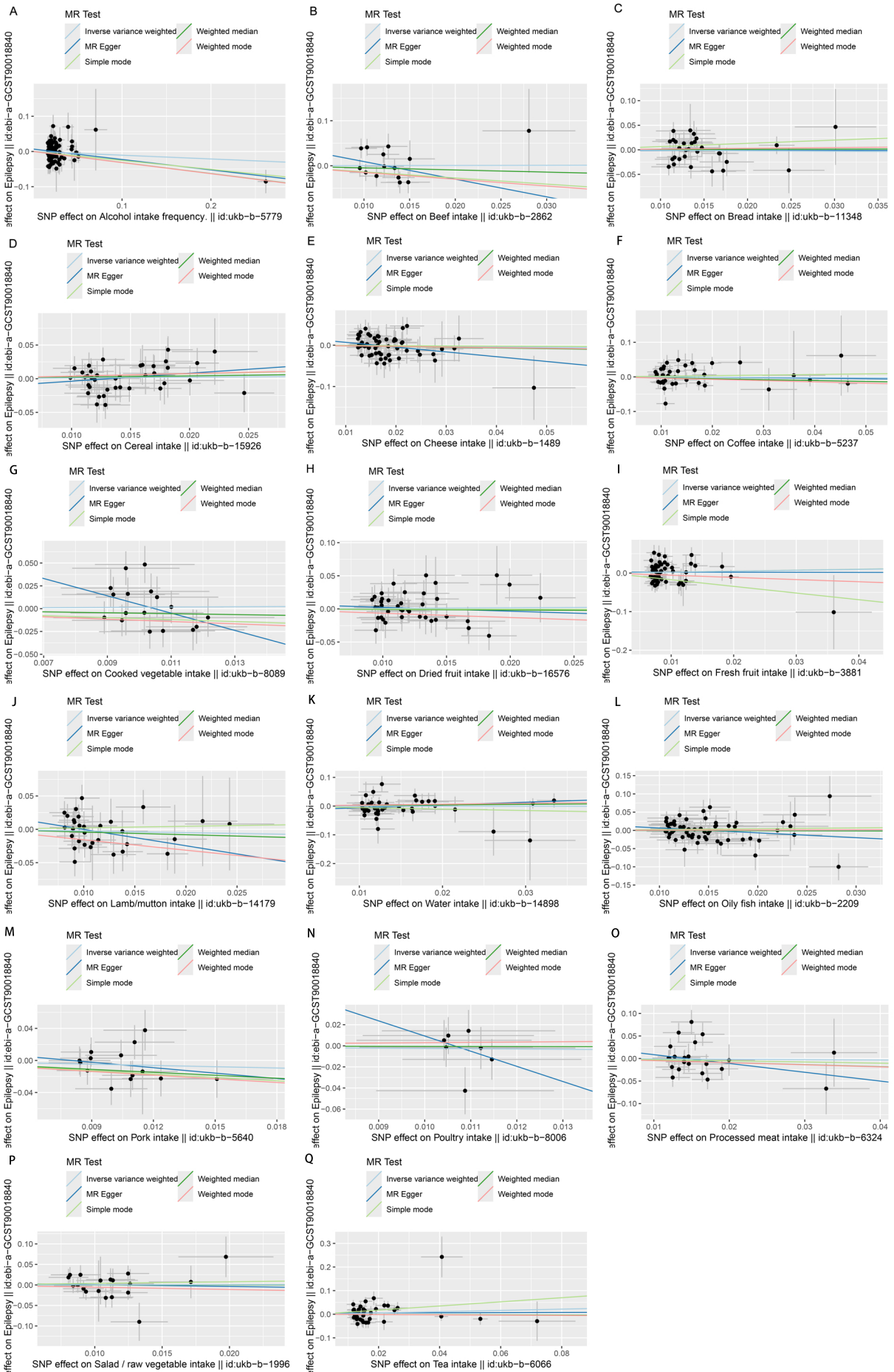

Supplement: Supplementary Figure S1 — MR leave-one out sensitivity analysis for dietary intake factors on epilepsy. [file Data_Sheet_2.zip › Supplementary Figure S2.pdf]

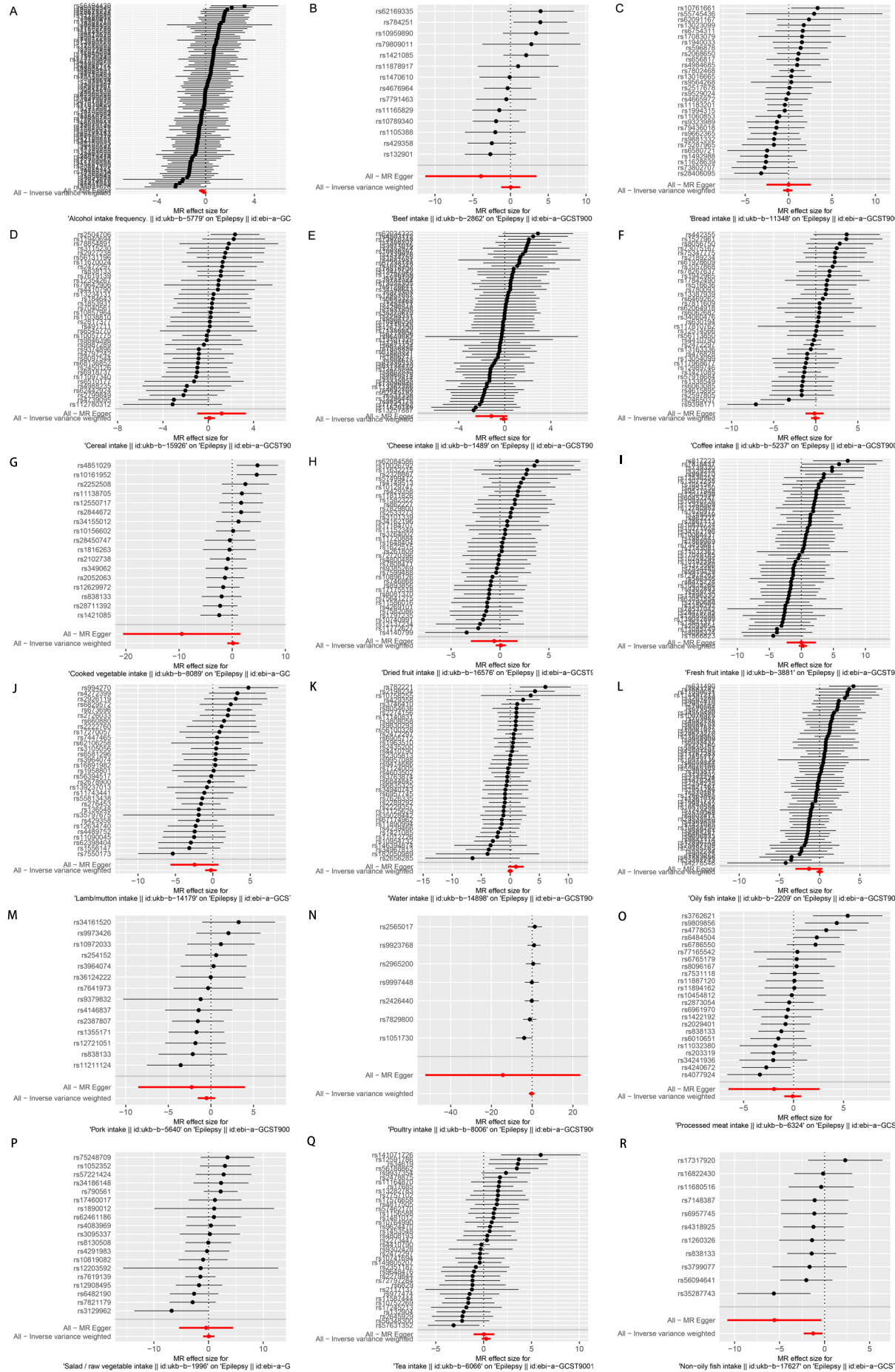

Supplement: Supplementary Figure S1 — MR leave-one out sensitivity analysis for dietary intake factors on epilepsy. [file Data_Sheet_2.zip › Supplementary Figure S3.pdf]

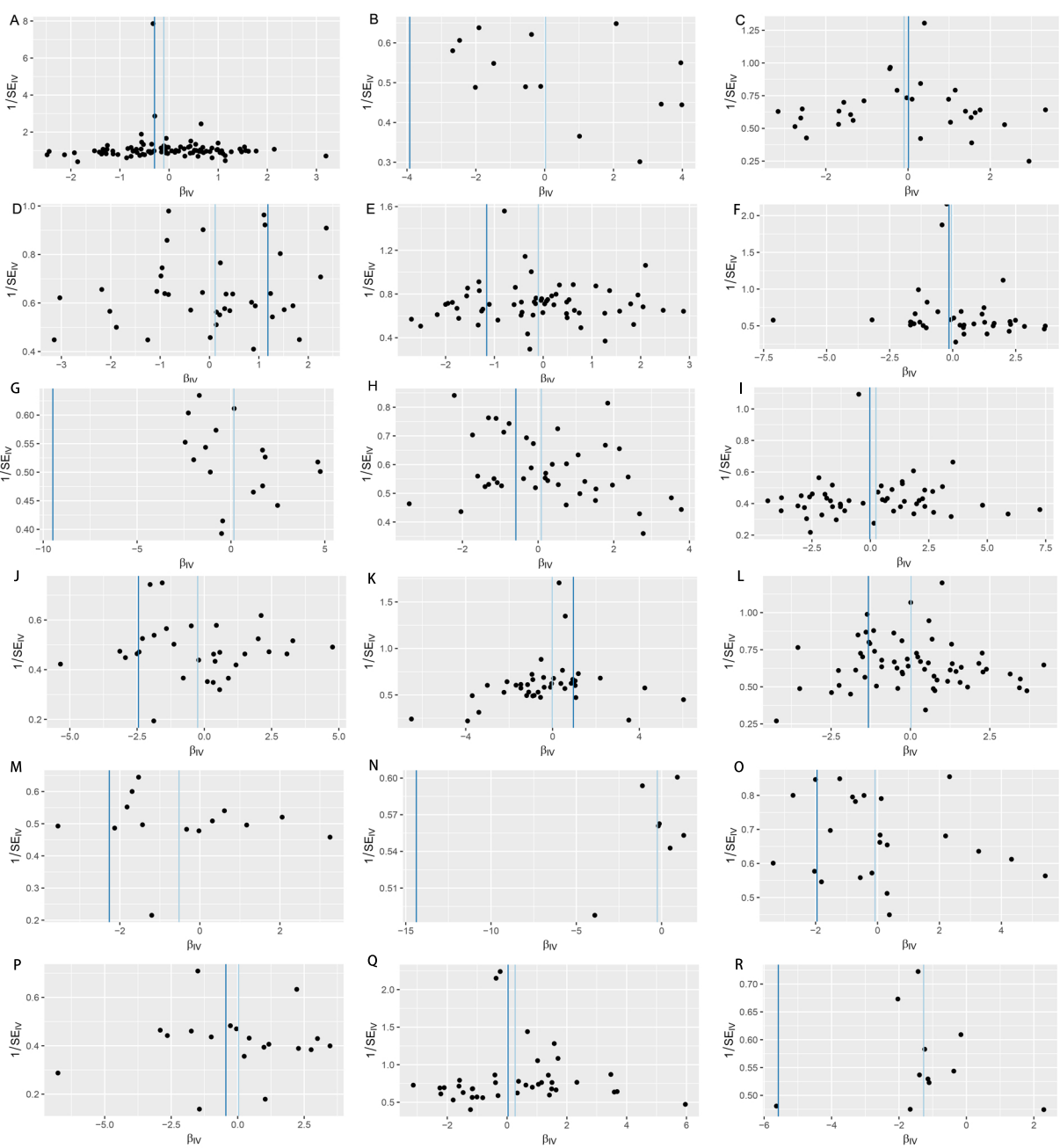

Supplement: Supplementary Figure S1 — MR leave-one out sensitivity analysis for dietary intake factors on epilepsy. [file Data_Sheet_2.zip › Supplementary Figure S4.pdf]
